# Exploring the Mediating Role of Executive Function in the Relationship between Aerobic Fitness and Academic Achievement in Adolescents

**DOI:** 10.3390/brainsci13040614

**Published:** 2023-04-04

**Authors:** Seyun Park, Haeyong Chun, Jennifer L. Etnier, Daehyun Yun

**Affiliations:** 1 Department Sport Science, Chungnam National University, 99 Daehak-ro, Yuseong-gu, Daejeon 34134, Republic of Korea; 2 Department of Kinesiology, Michigan State University, 109C IM Sports Circle Building 308 W. Circle Drive, East Lansing, MI 48824, USA; 3 Department of Kinesiology, University of North Carolina at Greensboro, Greensboro, NC 27402, USA

**Keywords:** physical activity, physical fitness, aerobic fitness, cognition, executive function, academic achievement, mediation, adolescent

## Abstract

(1) Background: The performance of physical activity at a sufficient volume can result in improvements in fitness. Aerobic fitness is a particular aspect of fitness that has consistently been shown to be related to both cognitive performance and academic achievement. Cognitive performance, particularly executive function, is itself predictive of academic achievement. It has been hypothesized that the benefits of aerobic fitness for academic achievement are due to improvements in executive function. This study explores the mediating role of cognitive performance on the relationship between physical fitness and academic achievement in high-school-aged adolescents. (2) Methods: High school students (N = 283, 127 male, 156 females, mean age = 16.05 years, SD = 0.41) performed a shuttle run test to assess aerobic fitness and completed the Stroop Color, Stroop Word, and Stroop Color–Word tests to measure information processing and inhibition. They also completed the National Union Academic Achievement Assessment (NUAAA) as part of their high school requirements. (3) Results: Mediation analyses showed that inhibition (performance in the Stroop Color–Word test) fully mediated the relationship between aerobic fitness and both mathematics and Korean performance. (4) Conclusions: This cross-sectional investigation suggests an important mediating role of cognitive performance related to executive function in understanding the relationship between aerobic fitness and the academic achievement of high-school-aged adolescents. This suggests that enhancements in performance related to executive function, which are attributed to increases in aerobic fitness, could explain the observed benefits for academic attainment.

## 1. Introduction

Many studies have reported a positive association between fitness and academic achievement in children [1,2,3] and adolescents [4,5,6,7,8]. Findings from a systematic review suggest that aerobic fitness is the most studied aspect of fitness in this research area, and is also the factor most consistently resulting in significant benefits to cognitive performance and academic achievement [9,10,11]. Further, meta-analytic reviews indicate that aerobic fitness has a moderately sized positive relationship with academic achievement [12].

Aerobic fitness is the capacity to transport oxygen to the muscles and employ it for energy production during physical activity. It is widely recognized as the most effective indicator of the cardiorespiratory system’s functional ability [13]. Furthermore, physical activity (PA) plays a critical role in enhancing aerobic fitness among children and adolescents. Systemic reviews and meta-analyses of physical activity interventions have demonstrated that they enhance physical fitness and aerobic capacity among youth aged 6–18 [14] and 10–19 years [15]. Being related to better academic achievement, aerobic fitness has also been shown to have a positive relationship with cognitive performance. The positive association between aerobic fitness and cognitive performance in children has been investigated, with meta-analytic results supporting an association between aerobic fitness and cognitive performance [9] and a positive effect for PA interventions on cognitive performance [16]. These latter findings support the hypothesis that PA benefits academic achievement through physical fitness and cognitive performance improvements. In a cross-sectional study with children (6–9 years), it was found that PA indirectly influenced academic achievement in the area of mathematics through the mediating effects of aerobic fitness [17]. Another study found a positive relationship between changes in aerobic fitness among adolescents and changes in academic achievement in reading and mathematics [18]. Furthermore, Sardinha et al. [8] followed 1286 adolescents aged 11–14 years for a period of 3 years and found a prospective association between consistently high or improving aerobic fitness and better academic achievement.

Considering the relationship between cognitive performance and academic achievement, diverse studies persistently report a positive relationship between one particular aspect of cognitive performance, executive function, and academic achievement [19]. This relationship has been shown to exist across cultures, with correlations evident for various aspects of executive function (inhibition, working memory, and attentional control) in Chinese and American preschool-aged children [20]. These relationships are consistently demonstrated across a range of studies [19,21,22]. In the case of adolescents, a recent study identified the significance of aerobic fitness in enhancing inhibitory control and cognitive flexibility [23], and suggested it was positively related to cortical brain development [24] and white matter [25]. During adolescence, white matter continues to develop, and its characteristics have been demonstrated to be essential for cognitive functions, such as executive function, as well as behavior, including risk-taking behaviors [25]. Additionally, the development of the cortical brain is associated with the enhancement of executive function [26].

Considering physical activity, aerobic fitness, cognitive performance, and academic achievement simultaneously, a systematic review exploring mechanisms of the PA–academic achievement relationship found that aerobic fitness and cognitive performance were consistently significant mediators of the relationship in empirical studies [27]. This review provides a basis for the hypothesis that improvements in aerobic fitness in adolescence will be able to enhance cognitive performance and academic achievement, and suggests that cognitive performance is predictive of academic achievement. In particular, the mediating role of executive function has recently attracted attention in this relationship [28,29]. Given the similarity between the brain regions where inhibitory processes operate and those associated with mathematics and reading performance [30], it is expected that inhibition will play a significant role in the relationship between aerobic fitness and academic achievement. However, the role of cognitive performance as a mediator of the effects of aerobic fitness on academic achievement has not been dealt with sufficiently for two reasons. The first relates to the age of the participants and the second to using latent variables to represent cognitive performance.

Recent studies found a mediating effect of cognitive function [31] and executive function [32,33] in the relationship between physical fitness and academic achievement in primary school children. These studies all show that cognitive performance is a mediator of the relationship between physical fitness and academic achievement. However, these studies were limited in that they considered executive function or cognitive performance as a latent variable and hence were unable to determine the mediating effect of specific aspects of cognitive performance. Another limitation of the literature is that researchers have previously mainly focused on children in primary school [29,31,31,32,33]. There is a lack of research on adolescents, with only a small number of studies even exploring the relationship between physical fitness and cognitive performance, or physical fitness and academic achievement [34,35]. In fact, as a result of synthesizing reviews on chronic physical activity and academic achievement, cognition, and the brain in children and adolescents, reviewers consistently point to the lack of studies involving adolescents [36]. Understanding the mediating role of cognitive performance in the relationship between aerobic fitness and academic achievement in adolescents is important because this developmental stage is a period when academic performance is influential. Hence, this study aims to explore the mediating effects of two measures of cognitive performance (information processing and inhibition) on the relationship between aerobic fitness and academic achievement in adolescents.

## 2. Methods

### 2.1. Participants

The participants for this study included a total of 283 high-school-age adolescents (127 male, 156 females, mean age = 16.05 years, SD = 0.41) in South Korea, who did not report any physical or mental health problems. The sample size of this study was selected by referring to previous studies related to multi-mediated effect analysis [37,38]. All the participants were living in urban areas, and they attended general public high schools where differences in social economic status were unlikely to occur since they lived in similar residential regions. Prior to selecting the final study participants, six students (5 male, 1 female) were excluded after reporting they had not finished the National Assessment of Educational Achievement test. After approval by Chungnam National University’s Institutional Review Board (202203-SB-024-01), the study took place with the voluntary participation of the participants. Their parents submitted a consent form after receiving a description of the study.

### 2.2. Measures

#### 2.2.1. Aerobic Fitness

Aerobic fitness was measured with a 20 m shuttle run, which was performed based on the manual of the Physical Activity Promotion System (PAPS) in order to examine the physical ability of all elementary, middle, and high school students in South Korea. The 20 m shuttle run or “PACER” is probably the most widely used field test to estimate aerobic fitness [39]. The shuttle run is a physical fitness test that assesses an individual’s aerobic capacity by quantifying the number of shuttle runs completed back and forth over a 20-meter distance within a predetermined time frame. This evaluation is used to examine physical ability in a wide range of populations such as children, teenagers, soldiers, and even firefighters. The measurement was performed at gyms in the participants’ high schools. The researchers measured performance with the cooperation of physical education teachers. The shuttle run was completed with 10 students of the same gender, who commenced the test simultaneously upon receiving a signal. The PAPS manual from the Korean Ministry of Education suggests a 5-level evaluation of fitness test outcomes depending on gender and age. Based on the PAPS manual, this study classified shuttle run records into 5 levels (5 higher–1 lower), thus controlling for gender and age.

#### 2.2.2. Cognitive Performance

Cognitive performance was examined by using the Stroop Word, Stroop Color, and Stroop Color–Word tests. The Stroop test is used widely to evaluate versatile cognitive functions such as attention, processing speed, cognitive flexibility, and inhibition [40]. Inhibitory control is a core indicator of cognitive executive control that is indicative of self-regulatory abilities [41]. The Stroop test is an examination of reading words and colors as accurately and fast as possible. The Stroop Word test requires participants to read aloud words printed in black ink, the Stroop Color test requires participants to verbally identify the color of colored blocks printed without words, and the Stroop Color–Word test requires participants to verbally identify the color of the ink in which words are printed, not read out the colors that are written down. This study used the method suggested by Golden [42] that measured the number of correct responses within 45 s. Participants in the study were instructed to perform a paper-printed Stroop Test with oral responses, the researcher pointed out the errors when they were made, and participants were to correct their errors before proceeding. The Stroop Word, Stroop Color, and Stroop Color–Word tests reflect processing speed, and the Stroop Color–Word test measures inhibition [40,43].

#### 2.2.3. Academic Achievement

The academic achievement of high schoolers was assessed by the National Union Academic Achievement Assessment (NUAAA) of Korea. The NUAAA is a paper test that is administered by education offices across Korea four times a year during the first and second years of high school. This study used standard scores for Korean and mathematics in order to reflect linguistic and mathematical achievement. Standard scores have the advantage of reflecting the relative rankings among examinees, regardless of the subject level. The class teachers provided the scores of the NUAAA for each participant. Generally, most Korean high school students participate in taking the NUAAA, which is administered across a day, from 08:40 a.m. to 16:32 p.m. The Korean and mathematics tests are conducted in the morning, with Korean tested for 80 min in the first session and mathematics tested for 100 min in the second session.

### 2.3. Procedures

The researchers collected data from students, who wished to participate voluntarily, from two general high schools. In cooperation with the high school, the researchers conducted an aerobic fitness test and a cognitive test after obtaining consent from parents and assent from students. The researchers and research assistants visited the high schools and gathered the aerobic fitness measurements in cooperation with physical education teachers. The fitness tests were conducted in the high schools’ gymnasiums during physical education classes. Cognitive performance was measured with the cooperation of a physical education teacher and a class teacher, and was measured at a time when physical activity had not been performed in the previous 2 h. The Stroop tests were conducted more than 2 weeks after the aerobic fitness test had been completed. The researcher provided face-to-face instructions and then assessed Stroop test performance for about 6 min in a quiet classroom. Academic achievement was operationalized as the NUAAA Korean and mathematics scores. After aerobic fitness and cognitive performance measurements, the researchers were provided with the NUAAA score from the time point most proximal to these assessments. All data collection was completed in approximately four months.

### 2.4. Data Analysis

Before analyzing the data, the data were screened to test for skewness and kurtosis between −2 and +2 to determine if the data were normally distributed [44]. Descriptive statistics were examined, and correlation analysis was employed to identify associations between the variables. In addition, a parallel multiple mediator model was employed to investigate the role of cognitive performance in the relationship between aerobic fitness and academic achievement. To mitigate the influence of gender, a covariate adjustment was implemented for gender, given that Korean scores exhibit gender-based discrepancies. Bootstrap confidence intervals [45] were used to show the significance of the mediating effect because the method provides superior verification power compared with the Sobel test. In the context of analyzing cognitive function and academic performance as latent variables using structural equation modeling, the analysis provides the total indirect effect. However, it does not provide separate bootstrapping for the specific indirect effect. In essence, the application of structural equation modeling precludes the assessment of individual indirect effects, thus necessitating the use of a process macro to derive and analyze these effects [46] (p. 152). The data were analyzed using IBM SPSS v.26, and PROCESS Macro v. 3.4.1 was employed to test each mediating effect and the significance of the effects. All the analyses were conducted at the 0.05 significance level.

## 3. Results

Descriptive statistics and the results of correlation analyses between aerobic fitness, cognitive performance, and academic achievement are presented in Table 1. Aerobic fitness was positively correlated with Stroop Word (r = 0.15, *p* < 0.05), Stroop Color (r = 0.14, *p* < 0.05), Stroop Color–Word (r = 0.19, *p* < 0.01), and mathematics (r = 0.15, *p* < 0.05), but not with Korean. Positive correlations were observed among all indicators of cognitive performance and academic achievement, except for a negative correlation between aerobic fitness and Korean.

**Figure 1:**
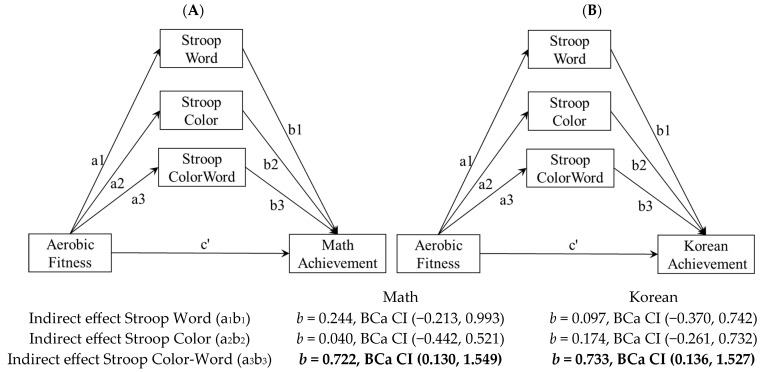
(**A**,**B**). Statistical diagram of the parallel multiple mediator model. Note: Confidence intervals presented are BCa bootstrapped, a1: the path from IV to SW, a2: the path from IV to SC, a3: the path from IV to SCW, b1: the path from SW to DV, b2: the path from SC to DV, b3: the path from SCW to DV, c’: the path from IV to DV.

Relationships between variables were analyzed by applying parallel multiple mediator models based on the significant associations among variables. The conceptual structural diagram of a parallel multiple mediator model is presented in Figure 1.

According to the results with mathematics as a dependent variable (Table 2), aerobic fitness was a significant predictor of Stroop Word (β = 1.55, *p* < 0.01), Stroop Color (β = 1.50, *p* < 0.05), and Stroop Color–Word (β = 2.05, *p* < 0.01) performance, while it did not significantly predict mathematics scores directly. Stroop Word and Stroop Color were not significant predictors of mathematics scores, but Stroop Color–Word was a significant predictor of mathematics scores (β = 0.35, *p* < 0.05). In testing the mediation effect of Stroop Word, Stroop Color, and Stroop Color–Word in the relationship between aerobic fitness and math, the indirect effect of Stroop Color–Word was significant (b = 0.722, BCa CI (0.130, 1.549)) while the indirect effects of Stroop Word and Stroop Color were not significant. These results revealed that Stroop Color–Word fully mediated the relationship between aerobic fitness and mathematics.

With Korean scores as the dependent variable (Table 3), aerobic fitness had a significant influence on Stroop Word (β = 1.55, *p* < 0.01), Stroop Color (β = 1.50, *p* < 0.05), and Stroop Color–Word (β = 2.05, *p* < 0.01); however, it did not significantly predict Korean scores. Stroop Word and Stroop Color were not significant predictors of Korean scores, but Stroop Color–Word (β = 0.36, *p* < 0.05) was a significant predictor of Korean scores. In testing the mediation effect of Stroop Word, Stroop Color, and Stroop Color–Word, only the indirect effect of Stroop Color–Word was significant (b = 0.733, BCa CI (0.136, 1.527)). These results revealed that Stroop Color–Word fully mediated the relationship between aerobic fitness and Korean.

## 4. Discussion

Studies investigating the positive effects of physical fitness or PA on cognitive performance and academic achievement in children and adolescents have increased since the 2000s, and have been expanding even more over the past decade [6,35]. The outcomes of these studies show that aerobic fitness (or cardiovascular fitness) is closely related to the executive function aspect of cognitive performance [1,12,47], and has a consistent positive relationship with academic performance in areas such as mathematics and linguistics [48]. Although the relationship has been consistently identified, not enough research has scrutinized the role of cognitive performance as a mechanism in the aerobic fitness–academic achievement relationship. In addition, the few studies on this subject have examined mainly primary school ages [10,35], while it seldom happens that research has explored the high school age group, where academic performance is important. Thus, this study investigated the mediating effect of cognitive performance in the aerobic fitness–academic achievement relationship in adolescents.

This study showed that cognitive performance in high-school-age adolescents had a full mediating effect on the relationship between aerobic fitness and academic achievement for both mathematics and linguistic performance. In short, aerobic fitness did not directly influence academic achievement, but rather had an indirect effect on it through the mediating role of cognitive performance. These results contribute to understanding the mechanism of the aerobic fitness–academic achievement relationship. According to relevant studies, aerobic fitness may impact structural and functional changes in the brain [49,50,51]. Some studies have shown that aerobic fitness is positively related to improving cognitive performance [20,52]. Therefore, a hypothesis has been raised about the mediating role of cognitive performance as a mechanism by which aerobic fitness affects academic achievement. The results of this study support this hypothesis, in that aerobic fitness had no direct influence on academic achievement, but demonstrated that aerobic fitness had an indirect positive effect on academic achievement through its relationship with cognitive performance.

In terms of aerobic fitness being a product of long-term PA, the findings that acute and chronic PA interventions affect cognitive performance and academic achievement improvement have meaningful implications. In this study, aerobic fitness individually had a positive effect on cognitive performance and, indirectly, on academic achievement. Reviews have shown that acute and chronic PA has a beneficial relationship with children’s brain health and fitness [53]. A recent study focusing on adolescents reported that acute PA in adolescents had a significant effect on the improvement of cognitive performance and academic achievement, and chronic PA also affected cognitive performance but had a greater effect on academic performance [54]. Recently, it was found that 20 min of acute aerobic PA improved EF in high school age groups (15.9 years old) [34]. This evidence suggests that PA can have a positive effect on cognitive function development and academic skills in high-school-age adolescents as well as in children, regardless of whether the PA occurs once or over the long term. This supports the importance of PA in adolescents’ daily lives. Previous research has shown that PA plays a significant role in improving aerobic fitness in children and adolescents [14,15]. Moreover, studies have demonstrated a positive relationship between adolescent aerobic fitness and brain development [24,26] as well as in the development of executive function [26] and academic achievement [8]. The findings of this study contribute to the expansion of this knowledge. However, few studies have explored the effect of chronic intervention in adolescents, so future studies are necessary [54].

A unique finding of this study was the individual mediating effect of each aspect of cognitive performance. The results showed that only inhibition (Stroop Color–Word) had a significant mediating effect, while the measures of information processing (Stroop Color, Stroop Word) did not have a mediating effect. This is consistent with the findings of a recent study that identified the mediating effects of executive function and aerobic fitness on the association between academic achievement and moderate to vigorous physical activity (MVPA) in 9- to 11-year-old children [27]. The authors showed that MVPA had a direct impact on academic achievement that was mediated by aerobic fitness and EF, so their findings are congruent with the results of this study. Additionally, the mediating effect of cognitive performance as a latent construct in the positive relationship between PA and academic achievement was demonstrated in primary school children [31,33]. However, it was not possible to identify the individual effects of each cognitive test because these studies employed SEM to verify the mediating effects.

The results of this study showed that inhibition had a significant mediating effect while information processing did not. These results suggest that inhibition, which is necessary for performance on the Stroop Color–Word test, fully explains the relationship between aerobic fitness and academic achievement. Although processing speed (Stroop Word and Stroop Color) is related to academic achievement, it does not explain the relationship between aerobic fitness and academic achievement for high-school-aged adolescents. This result is supported by a recent investigation with primary-school-aged children, which found that executive function fully mediated the relationship between aerobic fitness and academic achievement [32]. In addition, these results afforded evidence to reinforce existing findings [27,35] that showed the close relationship between aerobic fitness and academic achievement in children and adolescents.

Furthermore, it is noteworthy that this study dealt with academic achievement in a real-world setting. This study used grades from the NUAAA tests, which were conducted simultaneously across the nation. This study strengthened the validity of the research data by using comparable standardized scores of relative academic achievement.

Despite the contribution of the current study to extending knowledge on the relationships between the aerobic fitness, cognitive performance, and academic achievement of high-school-aged adolescents, this study has the following limitations. Firstly, the study measured cognitive performance related to information processing and inhibition using the Stroop Test. However, there are various methods to measure cognitive performance. Specifically, executive function, which is attracting attention in the relationship between aerobic fitness and academic achievement, is an umbrella term that includes at least three distinguishable attributes, i.e., shifting, updating, and inhibition, as well as elements such as working memory, cognitive flexibility, and planning [55]. Moreover, a recent investigation [29] reported that working memory and cognitive flexibility mediated the relationship between children’s cardiorespiratory fitness and mathematics and linguistics simultaneously, in addition to inhibition. Therefore, more diverse measurement batteries should be applied in future studies to measure adolescents’ executive function. Secondly, since this study was conducted using a cross-sectional design, there are limitations to confirming the causality of the relationships. To address this issue, intervention or longitudinal design studies are needed. Finally, although this study controlled for gender in the analysis, each variable may be influenced by demographic characteristics, individual interests, or motivation.

## 5. Conclusions

This study explored the relationships between physical fitness, cognitive performance, and academic achievement in high school adolescents, a population that has not been frequently considered. Additionally, this study tested the mediating effect of cognitive performance as a potential mechanism for the relationship between aerobic fitness and academic achievement. This cross-sectional study results showed that inhibition had a fully mediating effect on the relationship between aerobic fitness and academic achievement. Moreover, the results of this study are meaningful because of the assessment of academic performance in the real world. This will be valuable evidence to support the importance and necessity of enhancing PA in children and adolescents, and the provision of high-quality physical education classes and PA programs.

## Figures and Tables

**Table 1:** Means, standard deviations, and correlations of variables.

	Boys (*n* = 127)		Girls (*n* = 156)		Total (*n* = 283)		Correlations				
	M	SD	M	SD	M	SD	AF	SW	SC	SCW	Math
AF	3.84	1.02	3.00	1.92	3.38	1.05	1				
SW	91.92	8.96	92.31	8.41	92.13	8.65	0.149 *	1			
SC	74.09	12.30	72.97	10.90	73.48	11.54	0.135 *	0.589 **	1		
SCW	51.39	11.21	50.85	9.68	51.09	10.38	0.185 **	0.438 **	0.642 **	1	
Math	106.86	21.44	104.75	17.89	105.70	19.55	0.145 *	0.175 **	0.190 **	0.245 **	1
Korean	98.65	20.74	105.46	17.13	102.40	19.10	−0.055	0.153 **	0.198 **	0.241 **	0.665 **

Note. AF: Aerobic Fitness, SW: Stoop Word, SC: Stroop Color, SCW: Stroop Color–Word, Mathematics and Korean scores: standard scores 0~200, * *p* < 0.05, ** *p* < 0.01.

**Table 2:** Regression coefficients, standard errors, and model summary information for the parallel multiple mediator model with mathematics achievement.

	M1 (SW)			M2 (SC)			M3 (SCW)			DV (Math)		
	Coeff.	*SE*	*p*	Coeff.	*SE*	*p*	Coeff.	*SE*	*p*	Coeff.	*SE*	*p*
IV (AF)	_a1_ 1.55	0.53	<0.01	_a2_ 1.50	0.71	<0.05	_a3_ 2.05	0.63	<0.01	_c’_ 1.72	1.20	>0.05
M1 (SW)										_b1_ 0.16	0.16	>0.05
M2 (SC)										_b2_ 0.03	0.19	>0.05
M3 (SCW)										_b3_ 0.35	0.14	<0.05
CV (Gender)										−0.50	2.49	>0.05
Constant	84.29	2.98	<0.001	68.18	4.00	<0.001	42.34	3.56	<0.001	66.16	12.99	<0.001
	R^2^ = 0.03			R^2^ = 0.02			R^2^ = 0.04			R^2^ = 0.08		
	F (2, 280) = 4.37, *p* < 0.05			F (2, 280) = 2.59, *p* > 0.05			F (2, 280) = 5.37, *p* < 0.01			F (5, 277) = 4.51, *p* < 0.001		

Note. IV: independence variable, DV: dependent variable, CV: control variable, AF: Aerobic Fitness, SW: Stoop Word, SC: Stroop Color, SCW: Stroop Color–Word, a1: the path from IV to SW, a2: the path from IV to SC, a3: the path from IV to SCW, b1: the path from SW to DV, b2: the path from SC to DV, b3: the path from SCW to DV, c’: the path from IV to DV.

**Table 3:** Regression coefficients, standard errors, and model summary information for the parallel multiple mediator model with Korean achievement.

	M1 (SW)			M2 (SC)			M3 (SCW)			DV (Korean)		
	Coeff.	*SE*	*p*	Coeff.	*SE*	*p*	Coeff.	*SE*	*p*	Coeff.	*SE*	*p*
IV (AF)	_a1_ 1.55	0.53	<0.01	_a2_ 1.50	0.71	<0.05	_a3_ 2.05	0.63	<0.01	_c’_ −0.66	1.16	>0.05
M1 (SW)										_b1_ 0.06	0.16	>0.05
M2 (SC)										_b2_ 0.16	0.14	>0.05
M3 (SCW)										_b3_ 0.36	0.14	<0.05
CV (Gender)										6.55	2.40	<0.01
Constant	84.29	2.97	<0.001	68.18	4.00	<0.001	42.34	3.56	<0.001	61.90	12.54	<0.001
	R^2^ = 0.03			R^2^ = 0.02			R^2^ = 0.04			R^2^ = 0.10		
	F (2, 280) = 4.37, *p* < 0.05			F (2, 280) = 2.59, *p* > 0.05			F (2, 280) = 5.37, *p* < 0.01			F (5, 277) = 5.97, *p* < 0.001		

Note. IV: independence variable, DV: dependent variable, CV: control variable, AF: Aerobic Fitness, SW: Stoop Word, SC: Stroop Color, SCW: Stroop Color–Word, a1: the path from IV to SW, a2: the path from IV to SC, a3: the path from IV to SCW, b1: the path from SW to DV, b2: the path from SC to DV, b3: the path from SCW to DV, c’: the path from IV to DV.

## Data Availability

The data is unavailable due to privacy or ethical restrictions.
